# The “Whippets” of Paralysis: Galaxy Gas, Amazon, and the Deceptive GBS Mimic

**DOI:** 10.51894/001c.165689

**Published:** 2026-07-31

**Authors:** Adam Y. Makki, Philip Stone, Ajay Ghotra, Malaz Almsaddi, Jessica Scheuerman, Carrie Palazzolo, Abdullah A. B. Bokhari

**Affiliations:** 1 McLaren - Oakland

## 97

### Introduction

Nitrous oxide (N₂O) misuse is an increasingly recognized cause of neurologic injury, especially among young adults. Chronic intermittent exposure can cause functional vitamin B12 deficiency, leading to subacute combined degeneration (SCD) of the spinal cord. Early symptoms—paresthesias, gait instability, and weakness—are often nonspecific, and initial imaging may be normal. As a result, patients may be misdiagnosed with conditions such as Guillain-Barré syndrome (GBS), multiple sclerosis, or spinal cord compression. Early recognition in the emergency department is critical, as neurologic injury is potentially reversible with prompt treatment.

### Case Description

A 39-year-old incarcerated woman presented with two weeks of progressive bilateral lower extremity numbness, ascending paresthesias, intermittent weakness, and gait imbalance. She denied trauma, infectious symptoms, or bowel/bladder dysfunction. History was notable for alcohol use and chronic intermittent nitrous oxide (“whippets”) use over several years. On examination, cranial nerves were intact and upper extremity strength was normal. She had difficulty sustaining antigravity movement in the lower extremities, preserved deep tendon reflexes (2+), impaired two-point discrimination, and a positive Romberg sign. Laboratory evaluation showed mild macrocytic anemia (MCV 102) with a “low-normal” serum B12 level (310 pg/mL). CT head, MRI of the cervical/thoracic spine were unremarkable. Lumbar puncture revealed elevated cerebrospinal fluid protein without pleocytosis (albuminocytologic dissociation), raising concern for GBS. Given her substance use history and preserved reflexes, functional B12 deficiency with early SCD due to nitrous oxide toxicity was suspected. She was treated empirically with daily intramuscular vitamin B12 (1,000 mcg), admitted for further evaluation and counseling, and showed gradual neurologic improvement, with significant recovery at follow-up.

### Discussion

Nitrous oxide oxidizes and inactivates vitamin B12, impairing methionine synthase and myelin synthesis. This produces dorsal column dysfunction and macrocytic anemia through the “folate trap”, even when serum B12 appears normal or low-normal. Elevated methylmalonic acid and homocysteine can help confirm the diagnosis. N₂O toxicity can mimic GBS, particularly with elevated CSF protein; however, preserved reflexes and absence of autonomic instability favor a metabolic cause. Because imaging and laboratory findings can mislead early, diagnosis relies on clinical suspicion and targeted history. Prompt parenteral B12 therapy is essential to prevent permanent neurologic deficits.

